# Ethics of Health Data Infrastructures: Toward Continuous Governance and Public Trust

**DOI:** 10.2196/104402

**Published:** 2026-09-08

**Authors:** Yusuke Inoue, Jennifer Viberg Johansson

**Affiliations:** 1 Department of Healthcare Ethics, Kyoto University School of Public Health, Yoshida-Konoe-cho, Sakyo-ku, Kyoto, 606-8501, Japan, 81 75-753-4647; 2 Centre for Research Ethics & Bioethics, Department of Public Health and Caring Sciences, Uppsala University, Uppsala, Sweden; 3 Health Services Research, Department of Public Health and Caring Sciences, Uppsala University, Uppsala, Sweden

**Keywords:** health data infrastructures, governance, public trust, data stewardship, biobank, ethics, bioethics, research

## Abstract

In April 2026, reports of deidentified UK Biobank participant data being listed on overseas commercial online platforms highlighted concrete vulnerabilities in health data governance. In this viewpoint, we use the incident as an illustrative case to argue that traditional, trust-based, preaccess review models have ethical and operational limitations. The core concern is not data sharing, commercial involvement, or international collaboration per se, but unauthorized downstream movement of participant-contributed data beyond approved research governance into external commercial digital environments. We advance 3 key messages. First, governance should extend beyond initial access approval to continuous, proportionate stewardship across the data life cycle. Second, technical safeguards, including trusted research environments and audit logging, must be linked to institutional accountability for downstream data use. Third, public trust and social license require transparent communication, public-facing accountability, and governance mechanisms that remain responsive after access has been granted. As initiatives such as the European Health Data Space develop, legal alignment should be accompanied by operational accountability for downstream use. Continuous governance should complement, rather than replace, existing access review and research governance by maintaining proportionate oversight and accountability after access has been granted. This approach requires attention not only to technical safeguards but also to institutional responsibilities, implementation feasibility, and transparent communication with participants and publics. Health data infrastructures can sustain scientific value and public trust only when responsible data sharing is coupled with continuous, practical, and publicly accountable stewardship.

## Introduction

In April 2026, the UK authorities and UK Biobank reported that deidentified participant data from UK Biobank had been listed for sale on overseas commercial online platforms [1,2]. According to public statements, the listings were removed shortly after their discovery; no sale was believed to have occurred, and the data were described as not containing directly identifying information, such as names, addresses, contact details, telephone numbers, or NHS numbers [1,2]. Based on public statements, the incident appears to have involved unauthorized downstream redistribution or attempted sale of deidentified participant data after approved research access had been granted and therefore could not have been prevented through preaccess review alone.

UK Biobank subsequently suspended institutional access to data, paused access to its research platform, and announced additional security measures alongside a board-led investigation [1-3]. Official responses framed the incident as a matter not only of data security but also of governance, transparency, accountability, and public confidence in health data research [4,5].

Although UK Biobank is best known as a major infrastructure for genomic and biomedical research, this event has implications for a wider range of health data infrastructures, including disease registries, longitudinal cohort studies, and routinely collected health data linkage [6]. If public trust deteriorates, participation, data sharing, representativeness, long-term follow-up, and social acceptance of linkage may all be affected [7].

While caution is warranted in drawing conclusions from a single incident, recent years have seen growing international debate concerning reidentification risks, secondary use of health data in commercial AI development, cross-border data sharing, and governmental access to research datasets. This case illustrates that these concerns are no longer merely theoretical but may emerge as concrete operational challenges within real-world health data infrastructures. We use this incident as an illustrative case rather than as the sole empirical basis for broad claims about all health data infrastructures. This Viewpoint aims to clarify why existing governance should be strengthened beyond its predominant focus on preaccess review and to outline practical, proportionate mechanisms for stewardship before, during, and after data access. It is intended for those involved in governing, funding, operating, and using health data infrastructures, including data custodians, researchers, funders, regulators, technology providers, and participant or public representatives.

## From Access Review to Continuous Governance

A notable feature of this incident is that datasets formally provided through authorized governance procedures appeared on commercial online platforms outside conventional research governance frameworks [1,2]. The incident, therefore, does not indicate an absence of existing review and access systems. On the contrary, UK Biobank access is embedded within a wider governance structure involving research ethics review, confidentiality oversight, institutional vetting, and contractual controls [4]. However, this case raises questions about whether such preaccess mechanisms remain adequate once deidentified participant data move beyond institutional research environments and into external commercial spaces where conventional academic oversight no longer functions effectively.

The issue should not be understood solely as a conventional data breach, cybersecurity intrusion, or accidental exposure; rather, it raises questions of contractual or policy noncompliance and unauthorized downstream redistribution after approved access. Although existing governance includes contractual obligations and other postaccess controls, the governance of research data has historically focused predominantly on preaccess review. This incident highlights the need to strengthen the enforceability and implementation of continuous governance after data provision. We define continuous governance as active stewardship in which oversight, technical safeguards, accountability, and response capacity remain in place across the data life cycle, rather than ending once access has been approved. In practice, this includes monitoring data use, supporting user accountability, and responding to emerging risks after data access has been granted.

## Continuous Governance in Practice

Continuous governance requires oversight throughout the data life cycle rather than at a single point in time. To illustrate how this can be operationalized in practice, we focus on 3 interrelated dimensions: postaccess governance and safeguards, downstream data use, and public communication and trust. The following sections examine each of these perspectives in turn.

### Postaccess Governance and Safeguards

Open science has made data sharing an institutional priority in many countries. Participant-contributed health data are essential for medical research but generate social value only when used responsibly. Health data infrastructures, therefore, depend on public confidence that the entrusted data will be handled appropriately and for socially legitimate purposes. If that confidence is undermined, the issue is not limited to ethics or privacy; it may also weaken the legitimacy and sustainability of health data infrastructures.

The responsibilities of data access committees and custodians should be understood as extending beyond the initial authorization of access. As biobanks and registries have become increasingly central to medical research, governance responsibilities associated with data access decisions have become more important. Nevertheless, compared with research ethics review systems, the normative principles and institutional frameworks governing data access reviews remain relatively underdeveloped [8]. In particular, limited mechanisms exist for verifying how datasets are handled after access is granted or for intervening effectively when problematic downstream activities occur.

Technical governance mechanisms have become increasingly important. Trusted research environments (TREs) and data safe haven models, wherein researchers access secure analytic environments without exporting raw datasets, are likely to play a larger role in biobanks and registries [9]. The requirements for usage logs, third-party audits, and periodic compliance reviews could help strengthen the oversight of downstream data use after distribution. These technical safeguards can make inappropriate use more visible and easier to investigate, but they must be linked to institutional responsibilities for investigation, response, and public communication.

Concurrently, overly complex, slow, or poorly integrated governance mechanisms within routine research workflows can inadvertently foster insecure workarounds, such as local storage, informal file transfers, and the utilization of unmanaged environments. Empirical studies indicate that researchers frequently perceive existing governance systems as administratively burdensome and inadequately aligned with practical research realities [10]. Consequently, future health data infrastructures must prioritize security while maintaining sufficient usability to support ordinary research practice. Human-centered design in TREs transcends mere technical convenience; it facilitates appropriate data use as the default. The challenge extends beyond simply imposing stricter restrictions to embedding ethics by design within routine research environments [11,12].

Proportionality is central to this approach. Low-risk, routine uses should be supported through streamlined review and reusable secure workflows, whereas uses involving heightened risks, which may include certain cross-border transfers, highly identifiable linkage, unapproved commercial reuse, or model training, should trigger enhanced oversight. These examples are illustrative, and the appropriate level of oversight should reflect the nature of the data, the conditions of their use and transfer, and the feasibility of effective postaccess control. Proportionate stewardship, therefore, requires shared infrastructure, clear resourcing, and capacity building so that stronger governance does not become an unnecessary barrier to responsible research.

### Downstream Data Use

Continuous governance must also address the trajectory of research-derived data once they move beyond conventional academic research environments. Historically, the sharing of participant-contributed health data was primarily envisioned within relatively bounded academic communities governed by overlapping systems of ethics reviews, institutional oversight, journal policies, and research funding requirements. However, recent advances in data science and AI have expanded the potential uses of health data across a wide range of institutional settings, extending far beyond traditional academic research. Health-related data may traverse clinical, research, commercial, consumer, and public health contexts, in which privacy protections and ethical oversight differ substantially.

As a result, participant-contributed data may be reused or linked in ways that fall outside the assumptions of conventional research ethics governance [13]. Recent legislative debates in Japan also illustrate the expanding policy interest in the secondary use of data for statistical analysis and AI development [14]. Generative AI and large language model development may intensify this challenge because removing the influence of data used in model training can be technically difficult [15].

This cross-sectoral circulation represents more than a technical boundary crossing. It raises questions about whether the expectations and safeguards that sustain participants’ altruistic trust can endure as data traverse different institutional settings. Commercial actors, alongside other nonacademic entities, are often essential in translating research into medical and technological innovations. The ethical issue is not commercial involvement itself; approved commercial collaboration under public-benefit conditions should be distinguished from unauthorized onward transfer, data brokerage, or platform-based resale outside approved governance. Tensions arise when data contributed under expectations of public benefit move into downstream contexts shaped by differing norms, incentives, and accountability mechanisms, particularly when there is insufficient societal accountability or transparency.

In such situations, participants may perceive an imbalance between their contributions and the purposes, actors, or benefits that their data ultimately support, generating concerns regarding fairness, reciprocity, and goal displacement. These dynamics can weaken the moral foundation on which large-scale health data infrastructures depend.

The circulation of research data beyond conventional academic governance environments cannot be reduced to individual contractual violations; rather, it raises broader questions about whether internal academic self-governance alone is sufficient when research-derived data are reused, linked, or transferred across institutional and, in some cases, national borders [8]. In some circumstances, coordination with regulatory authorities and cross-border data access mechanisms may therefore be necessary. Sweden’s amended Biobank Act illustrates this approach by linking access to identifiable biobank samples for research with ethics approval and biobank-level conditions governing purpose, transfer, analysis, and post-study handling [16]. The European Health Data Space Regulation offers an emerging EU-level model for such health data infrastructure. Although still in a transitional phase, it is intended to enable secure secondary use of electronic health data through common access bodies, data permits, cross-border infrastructure, and secure processing environments [17,18].

At the same time, regulatory coordination should remain carefully bounded. Stronger oversight may be necessary to sustain trust in cross-border health data infrastructures. However, it should not become a mechanism for disproportionate governmental access, nor should it unduly constrain multidisciplinary collaboration and the independent development of research infrastructures [19]. The aim is therefore not a uniform restriction of data sharing, but a multilayered governance framework that clarifies the roles, responsibilities, and limits of the actors involved. Table 1 illustrates the complementary roles of key actors within such a framework.

**Table 1.** Illustrative roles and responsibilities in continuous governance.

Actor or governance layer	Primary role	Illustrative examples
Participants and publics	Provide perspectives on societal expectations, needs, and concerns	Input on acceptable data uses, feedback on governance and communication
Health data custodians and infrastructure operators	Steward health data infrastructures across the data life cycle	Trusted research environments, postaccess oversight, transparent communication
Data access bodies	Enable proportionate and accountable access	Risk-based access review, review of higher-risk uses
Researchers and research institutions	Generate scientific value through responsible data use	Purpose-aligned data use, secure workflows
Commercial and other downstream users	Develop practical and innovative uses while complying with access conditions	Approved applications, compliance with access conditions, reporting emerging risks
Funders and regulators	Support and oversee proportionate governance	Shared infrastructure, capacity building, governance requirements

### Public Communication and Trust

Public communication about data protection and downstream data-use risks is another important dimension of continuous governance. In responding to the incident, UK Biobank emphasized that its data remained “safe and secure” and sought to reassure participants that the affected data were “deidentified” and contained no personally identifying information [2]. Such safeguards remain essential to biobank- and registry-based research. However, the incident also raises the question of whether reassurance focused primarily on identifiability adequately addresses participants’ concerns when data are redistributed or used in downstream contexts that were not originally anticipated. Ongoing debates concerning reidentification through data linkage and AI-based analysis further underscore the need for broader communication about downstream data-use risks [20,21].

The ethical concerns raised by downstream redistribution cannot be reduced solely to measurable risks of reidentification or direct harm. Researchers themselves have described tensions between maximizing societal benefit through broad data sharing and respecting individuals’ expectations regarding how their data circulate across institutional and commercial environments [10]. Even in situations where no confirmed misuse occurs, unexpected downstream redistribution of data may generate perceptions of loss of control, violations of trust, or misuse of institutional commitments. In Nissenbaum’s [22] terms, the issue also constitutes a disruption of expected contextual flows between participants, institutions, and downstream users. Such disruption raises questions of governance, transparency, legitimacy, and alignment between public expectations and real-world data practices.

In this case, the rapid public response by relevant organizations, including the removal of listings, suspension of associated access, and the subsequent UK Biobank Oversight Committee review, demonstrated recognition of the seriousness of the issue and an effort to maintain transparency with society [2,3]. The National Data Guardian [5] similarly emphasized that immediate steps to secure data must be matched by transparency, accountability, and clear answers for participants if public confidence in responsibly governed health data research is to be maintained.

Data infrastructures are not merely technical systems; they are public institutions whose long-term operation depends on continued public participation and acceptance. Here, public trust refers to confidence placed in institutions, whereas trustworthiness refers to the practices and accountability structures that merit such confidence. Legitimacy pertains to whether data practices can be publicly justified, and social license refers to continuing public acceptance over time, rather than a one-time permission secured at recruitment [23]. Social license may therefore be supported by routine opportunities for public input and public justification, including participant or citizen advisory groups, public reporting, and other participatory mechanisms. Such engagement can support legitimate data sharing rather than merely restrict it.

## Conclusions

This UK Biobank case should be regarded as an opportunity to reconsider the governance of health data infrastructures without retreating from responsible data sharing. Excessive restrictions, such as blanket data localization requirements that prevent responsible cross-border research, should be avoided. At the same time, governance models relying solely on trust-based assumptions are unlikely to be sufficient when participant-contributed data can circulate across decentralized, commercial, and international digital environments. These priorities are especially relevant as initiatives such as the European Health Data Space make cross-border secondary use more routine [17,18]. This implies 4 practical priorities. First, health data infrastructure custodians and data access bodies should have postaccess powers, including use reports, audit logs, controlled output release, risk-based review, and temporary suspension during investigations. Second, funders and regulators should require downstream governance plans as part of approval or funding conditions. Third, infrastructure operators and technology providers should make TREs and data safe havens secure but usable, with limits on raw data export and support for accountable analysis. Fourth, participant- and public-facing transparency, including public reports or dashboards on approved users and purposes, should become routine.

Continuous governance must also be implemented with attention to feasibility and equity. Secure processing environments, audit systems, and transparency tools require resources that vary across institutions and countries. Implementation should therefore support shared infrastructure, capacity building, and proportionate approaches without creating unnecessary barriers to responsible research. Health data infrastructures can sustain both scientific value and public trust only when data sharing is matched by continuous, practical, and publicly accountable stewardship.

## Abbreviations

TRE: trusted research environment

## References

[1] (2026). UK Biobank data. Hansard - UK Parliament.

[2] Collins R (2026). A message to our participants: UK Biobank data security update. UK Biobank.

[3] (2026). Oversight Committee report into data security at UK Biobank published. UK Biobank.

[4] (2026). Our response to concerns over UK Biobank data. Health Research Authority.

[5] National Data Guardian (2026). National Data Guardian statement on UK Biobank data advertised for sale in China. GOV.UK.

[6] Sudlow C, Gallacher J, Allen N (2015). UK Biobank: an open access resource for identifying the causes of a wide range of complex diseases of middle and old age. PLoS Med.

[7] Harron K, Dibben C, Boyd J (2017). Challenges in administrative data linkage for research. Big Data Soc.

[8] Samuel G, Lucassen A (2023). Access to biobanks: responsibilities within a research ecosystem. Biopreserv Biobank.

[9] Jones KH, Ford DV, Jones C (2014). A case study of the Secure Anonymous Information Linkage (SAIL) Gateway: a privacy-protecting remote access system for health-related research and evaluation. J Biomed Inform.

[10] Viberg Johansson J, Bentzen HB, Mascalzoni D (2022). What ethical approaches are used by scientists when sharing health data? an interview study. BMC Med Ethics.

[11] Bygrave LA (2017). Data protection by design and by default: deciphering the EU’s legislative requirements. Oslo Law Rev.

[12] Brey P, Dainow B (2024). Ethics by design for artificial intelligence. AI Ethics.

[13] Konnoth C, Solaiman B, Cohen IG (2024). Research Handbook on Health, AI and the Law.

[14] (2026). Regarding the Bill to Amend the Act on the Protection of Personal Information and Other Acts [Article in Japanese]. Personal Information Protection Commission Japan.

[15] Liu S, Yao Y, Jia J (2025). Rethinking machine unlearning for large language models. Nat Mach Intell.

[16] Viberg Johansson J, Fredriksson M (2026). Implementing Sweden’s Biobank Act (2023:38): insights and lessons for international collaboration in health research. Biopreserv Biobank.

[17] Official Journal of the European Union (2025). Regulation (EU) 2025/327 of the European Parliament and of the Council of 11 February 2025 on the European Health Data Space and amending directive 2011/24/EU and regulation (EU) 2024/2847. EUR-Lex: EU Law.

[18] (2025). European Health Data Space Regulation (EHDS). European Commission.

[19] Arnason V (2004). Coding and consent: moral challenges of the database project in Iceland. Bioethics.

[20] Rocher L, Hendrickx JM, de Montjoye YA (2019). Estimating the success of re-identifications in incomplete datasets using generative models. Nat Commun.

[21] Gymrek M, McGuire AL, Golan D, Halperin E, Erlich Y (2013). Identifying personal genomes by surname inference. Science.

[22] Nissenbaum H (2004). Privacy as contextual integrity. Wash Law Rev.

[23] Carter P, Laurie GT, Dixon-Woods M (2015). The social licence for research: why care.data ran into trouble. J Med Ethics.

